# Inducible Resistance to β-Lactams in Oxacillin-Susceptible *mecA1*-Positive *Staphylococcus sciuri* Isolated From Retail Pork

**DOI:** 10.3389/fmicb.2021.721426

**Published:** 2021-10-20

**Authors:** Yifei Cai, Liangjun Zheng, Yao Lu, Xu Zhao, Yanting Sun, Xingyuan Tang, Jinhe Xiao, Chen Wang, Chao Tong, Lili Zhao, Yingping Xiao, Xin Zhao, Huping Xue

**Affiliations:** ^1^College of Animal Science and Technology, Northwest A&F University, Xianyang, China; ^2^College of Veterinary Medicine, Henan Agricultural University, Zhengzhou, China; ^3^Institute of Quality and Standard for Agro-Products, Zhejiang Academy of Agricultural Sciences, Hangzhou, China; ^4^Department of Animal Science, McGill University, Sainte-Anne-de-Bellevue, QC, Canada

**Keywords:** β-lactams, *Staphylococcus sciuri*, resistance, wall teichoic acid (WTA), OS-MRS

## Abstract

Most isolated strains of *Staphylococcus sciuri* contain *mecA1*, the evolutionary origin of *mecA*, but are sensitive to β-lactams (OS-MRSS, oxacillin-susceptible *mecA1*-positive *S. sciuri*). In order to improve the efficacy of antibiotic treatment, it is important to clarify whether the resistance of OS-MRSS to β-lactams is an inducible phenotype. In this study, three OS-MRSS strains with oxacillin MIC = 1 μg/ml were isolated from 29 retail pork samples. The resistance of OS-MRSS to β-lactams (MIC > 256 μg/ml) was found to be induced by oxacillin, and the induced resistance was observed to remain stable within a certain period of time. Interestingly, the induced β-lactam resistance was not caused by *mecA1*, heterogeneous resistance, or any genetic mutation, but mainly due to increased wall teichoic acid (WTA) synthesis that thickened the cell wall. The induced strains also showed slower growth rate, as well as decreased adhesion ability and biofilm thickness. These phenotypes were found to be achieved through altered gene expression in associated pathways, such as the citrate cycle and pentose phosphate pathway. The results challenge the traditional antibiotic sensitivity test. In the presence of β-lactam antibiotics, OS-MRSS that was initially sensitive to β-lactams was observed to gradually develop β-lactam resistance in several days. This often-neglected phenomenon in antibiotic sensitivity tests requires further research attention.

## Introduction

*MecA* gene that encodes a low-affinity penicillin-binding protein PBP2a, is a major determinant of β-lactam resistance in methicillin-resistant *Staphylococcus aureus* (MRSA). Several studies have suggested that *mecA1*, a close homolog of *mecA* found ubiquitously in *Staphylococcus sciuri* (*S. sciuri*) is the evolutionary origin of *mecA* (Rolo et al., 2017; Miragaia, 2018). Since *S. sciuri* is isolated from a wide range of animals as well as environmental samples (Kloos, 1980), it can be a potential threat to animals and public health. For instance, *S. sciuri* isolated from swine farms (Nemeghaire et al., 2014b; Kumar et al., 2018) has been found to cause fatal exudative epidermitis in piglets (Chen et al., 2007; Lu et al., 2017) due to exfoliative toxin C (ExhC) (Li et al., 2011). *S. sciuri* has also been found to be associated with infections in bovine (Vanderhaeghen et al., 2013; Nemeghaire et al., 2014b; Qu et al., 2019), poultry (Lu et al., 2020), and horses (Beims et al., 2016). Additionally, *S. sciuri* isolated from hospital environment (Dakic et al., 2005) has been implicated in case reports of peritonitis (Meservey et al., 2020), amputations (Coimbra et al., 2011), and other complications.

Compared with systematic studies of MRSA β-lactam resistance, research on *S. sciuri* antibiotic resistance is very scarce. Although there are only some sporadic reports (Couto et al., 2003; Stepanović et al., 2006; Rolo et al., 2017), there is a consensus that most isolated *S. sciuri* strains containing *mecA1* are sensitive to β-lactams (OS-MRSS) (Nemeghaire et al., 2014a; Rolo et al., 2017). However, some studies have found that the β-lactam resistance of oxacillin-susceptible *mecA*-positive *S. aureus* (OS-MRSA) can be induced by sub-inhibitory concentration of oxacillin due to upregulated *mecA* expression (Pilong et al., 2016). Hence, it is necessary to clarify whether the β-lactam resistance of OS-MRSS is inducible by oxacillin.

In this study, we found that oxacillin-induced β-lactam resistance existed in OS-MRSS and remained stable within a certain period of time. However, unlike OS-MRSA, this phenotype is not caused by *mecA1* or any genetic mutation, but mainly the increased synthesis of teichoic acid that thickens the cell wall. In view of its pathogenicity, the inducible resistance of OS-MRSS may be a significant potential threat to therapeutic efficacy of antibiotics.

## Materials and Methods

### Bacteria Strains, Plasmids, and Growth Conditions

Bacteria strains used in this study were isolated from retail pork. PCR was performed to investigate the presence of *mecA*, *mecA1*, *mecR1*, *mecI*, *blaR1*, and *blaI* genes (Supplementary Table 1). *MecA1* was inserted into pSE1 expression vector (Genbank no. KY615710) to construct pSE1-*mecA1*, which was subsequently electroporated into *S. aureus* Newman and RN4220, respectively. All key bacteria strains and plasmids used in this study are shown in Table 1. *S. sciuri* and their induced derivatives were grown in tryptic soy broth (TSB) medium, and their growth curves were recorded via OD_600_ measurement and viable bacteria count at 1-h interval for 20 h.

**TABLE 1:** Isolates and plasmids used in this study.

Isolates and plasmids	Characteristics	Sources
NWAF05	*S. sciuri, mecA1* ^+^	This study
NWAF25	*S. sciuri*, *mecA1*^+^	This study
NWAF26	*S. sciuri, mecA1* ^+^	This study
NWAF26^R^	The 10th generation for NWAF26 induced with oxacillin	This study
NWAF26^R^-R3	The 3th generation for NWAF26^R^ induced without oxacillin	This study
NWAF26^R^-R10	The 10th generation for NWAF26^R^ induced without oxacillin	This study
NEWMAN	*S. aureus, mecA^–^*, *mecA1*^–^, β-lactamase negative	Laboratory strain
RN4220	*S. aureus*, *mecA*^–^, *mecA1*^–^, β-lactamase negative	Laboratory strain
NEWMAN-*mecA1*	NEWMAN transformed with pSE1-*mecA1*	This study
RN4220-*mecA1*	RN4220 transformed with pSE1-*mecA1*	This study
pSE1	*S. aureus* expression vector, Apr, Cmr	This study
pSE1-*mecA1*	pSE1 with *mecA1* gene from isolate NWAF26	This study

### Antibiotic Susceptibility Tests

Spot assay was used to determine the minimal inhibitory concentration (MIC) of antibiotics in *S. sciuri* wild-type strains and their induced derivatives (Alex et al., 2014). In parallel, the strain survival rates were recorded in 30 ml of TSB medium supplemented with 20 mg/L of oxacillin (20-fold increase in wild-type MIC). Viable colonies were counted in cultures incubated on TSA plates. Bactericidal time with a survival rate of 1% (MDK99) was calculated according to the corresponding survival rate curve at five time points (0, 6, 12, 24, and 48 h).

### Antibiotic Induction and Reverse Induction Tests of *Staphylococcus sciuri*

Antibiotic induction was conducted by culturing *S. sciuri* NWAF26 and NWAF25 in TSB. Cultures grown without antibiotics were considered as passage 0 or day 0. Subsequently, batch cultures of *S. sciuri* were constantly exposed to sub-inhibitory gradient concentrations of oxacillin at 0, 0.5, 1, 2, 4, 8, 16, 32, 64, 128, and 256 mg/L from day 0 to day 10, respectively. Reverse induction was conducted by inoculating NWAF26^R^ in TSB without oxacillin. The culture was continuously transferred and passaged for 10 days without oxacillin. Reverse-induced bacteria on day 3 (NWAF26^R^-R3) and day 10 (NWAF26^R^-R10) were selected for subsequent tests.

### Whole-Genome Sequencing and Assembly

Whole-genome sequencing (WGS) was conducted on genomic DNA samples extracted from NWAF26 and NWAF26^R^ using Illumina paired-end 150 bp (PE150) (Illumina Novaseq 6000, Novogene, Beijing, China) and BEISEQ sequencing platform PE100 (BGI, Shenzhen, China) technologies. WGS sequence data were *de novo* assembled into contigs using SOAPdenovo software v.2.21, SPAdes, and Abyss. Furthermore, the contigs were joined into scaffolds using paired-end information. The order of the scaffolds was determined by alignment to a reference genome of *S. sciuri* strain B9-58B (GenBank no. CP041879) using SOAPaligner2 software. Gaps were closed by PCR and Sanger sequencing (Supplementary Table 1). The genome sequence of NWAF26 was deposited in GenBank. A map of the whole genome was generated by CGView. Antibiotic resistance genes were analyzed by searching the Comprehensive Antibiotic Resistance Database (CARD)^1^. Gene prediction and annotation were performed using the Rapid Annotations Subsystems Technology (RAST) server^2^.

### Transcriptome Sequencing and Bioinformatics Analysis

Total RNA of *S. sciuri* was extracted using the TRizol reagent (Sigma-Aldrich, United States) and Bacterial Total RNA Extraction kit (Sigma-Aldrich, United States) based on the instruction of the manufacturer. The quality and quantity of the RNA samples were determined by Ultramicro spectrophotometer K5500 (Beijing Kaiao Technology Development Co., Ltd., China) and Agilent 2200 TapStation (Agilent Technologies, United States). RNA-seq library construction and RNA sequencing were performed in biological triplicates using BGI-seq platform paired-end 150 bp (PE150) (BGI, Shenzhen, China). An average of 23.48 M RNA-seq reads per sample were obtained for analysis (21.53–25.11 M) and submitted to Sequence Read Archive (SRA) in NCBI (accession no. PRJNA754201). Reference genome sequences of *S. sciuri* NWAF26 and gene model annotation files were downloaded directly from the NCBI database (Genbank numbers: CP048732, CP048733, CP048734, and CP048735). Bowtie2-2.2.3 was used to build the index of the reference genome and align clean reads to the reference genome. HTSeq-0.6.1 was used to count the read numbers mapped to each gene, which were used to calculate the FPKM (fragments per kilobase million) values. Differential expression analysis of two conditions/groups was performed using the DESeq R package. After adjusting by BH (FDR correction with Benjamini/Hochberg) to control the false discovery rate, *p* < 0.05 and | log2FC| ≥ 1 were set to indicate significant differentially expressed genes (DEGs) (Ali et al., 2008). DEGs with at least one FPKM value >50 were selected for final analysis.

In order to validate the RNA-seq results, qRT-PCR (Supplementary Table 1) was performed to quantify the mRNA transcripts of 25 selected DEGs using the Light Cycler 480 (Roche, Switzerland) according to the instructions of the manufacturer (TransStart TipTop Green qPCR SuperMix, TransGen Biotech). Each qRT-PCR reaction was performed in a final volume of 25 μl. The thermal cycling profile was as follows: 42°C for 60 min and 72°C for 10 min; 40 cycles of 95°C for 15 s, 60°C for 30 s, 72°C for 30 s, and a final extension of 68°C for 10 min. Negative control samples containing sterile water were also included. The cycle threshold value (CT) was determined, and the relative fold difference was calculated by the 2^–△^^△^^CT^ method using 16S rRNA as the reference gene. Primer efficiency was predicted using Primer Premier 5 and calculated by analyzing the qRT-PCR dissolution curve. Each sample was determined in triplicate.

### Wall Teichoic Acid Isolation and Purification

Cell wall and WTA were isolated as described before (Peschel et al., 1999; Weidenmaier et al., 2004). In brief, a 20-ml culture of *S. sciuri* was cultivated overnight in TSB at 37°C, cells were collected by centrifugation, washed twice in sodium acetate buffer (20 mM, pH 4.7), and disrupted with glass beads. Cell lysates were incubated overnight with DNase I and RNase A at 37°C, and SDS extraction was performed. Subsequently, SDS and soluble cell components were removed by washing with sodium acetate buffer. The amount of cell wall was determined by weighing the cell wall after drying. WTA was extracted from purified cell walls using 5% trichloroacetic acid and incubating at 60°C for 4 h. The peptidoglycan was removed by centrifugation. WTA was quantified by testing the inorganic phosphate (Pi) content (Weidenmaier et al., 2004). The WTA isolation and Pi content assay were performed in triplicate for each strain.

### Transmission Electron Microscopy Analysis

Transmission electron microscopy (TEM) sample preparation processing of *S. sciuri* strains NWAF26, NWAF26^R^, and NWAF26^R^-R10 was conducted as described previously (Prufert, 2004). Cell wall thickness (CWT) was determined based on the thickness of 50 randomly selected cells per strain at five different spots on the cell wall. All selected cells had no septa to ensure that all cells were in the same stage of the cell cycle. Significance values of CWT were calculated using a two-tailed unpaired Student *t*-test.

### Adhesion of *Staphylococcus sciuri* to Host Cells

*Staphylococcus sciuri* adherence to host cells was determined by counting the CFUs of adhered bacteria (Ji, 2014). Briefly, MAC-T (bovine mammary epithelial cell) cells and Caco-2 (human colorectal cancer cells) cells were grown in 24-well culture plates. Following a washing step, approximately 10^7^ CFU of bacteria was added into each well. Non-adherent bacteria were washed off and released by Triton X-100. The mixture was inoculated on TSA plates for colony count. Adherence was calculated by dividing the original CFU of inoculum by the adhered CFU.

### Biofilm Formation Analysis

Biofilm production was analyzed using 96-well plate-based quantitative adherence assays as previous (Ansari et al., 2014). Biofilm formation was visualized by confocal laser scanning microscopy (CLSM) (Ansari et al., 2014). In brief, 200 μl of diluted bacteria was added into a glass-bottom culture dish (Nest) containing 2 ml of fresh GTSB medium. Biofilm was incubated at 37°C under static cultivation and fixed with 2.5% glutaraldehyde (Solarbio) for 1.5 h. Fixed biofilm was dyed with FITC-CONA and PI, sequentially washed with PBS and covered with antifade solution (Solarbio). The biofilm architecture was studied by using an inverted CLSM from Zeiss (Heidelberg, Germany). The biofilm intensity was determined by measuring the average fluorescence intensity of the scanning area using Image-Pro Plus 6.0 software.

## Results

### Staphylococci Are the Main Infectious Bacteria of Retail Pork

In order to investigate the pathogenic bacteria in retail pork, 29 samples of retail pork were sourced (20 from food markets and 9 from e-commerce platforms) in Hangzhou, China, from April 2017 to September 2017. Out of 52 pathogenic bacteria isolated and identified, 39 isolates (75%) were staphylococci and 13 isolates (25%) were *Enterococcus faecalis* (Table 2). Among the 39 staphylococci isolates, there were 27 *S. aureus* (including 4 MRSA), 5 *S. sciuri*, 5 *S. saprophyticus*, and 2 *S. pasteurii*.

**TABLE 2:** Investigated isolates from retail pork in food markets and e-commerce stores.

	*S. aureus*	*S. sciuri*	*S. saprophyticus*	*S. pasteuri*	*E. faecalis*
Food markets	23	3	2	2	11
E-commerce platforms	4	2	3	0	2

### Stable Resistance Against β-Lactam Antibiotics Can Be Inducted in *Staphylococcus sciuri*

**FIGURE 1:**
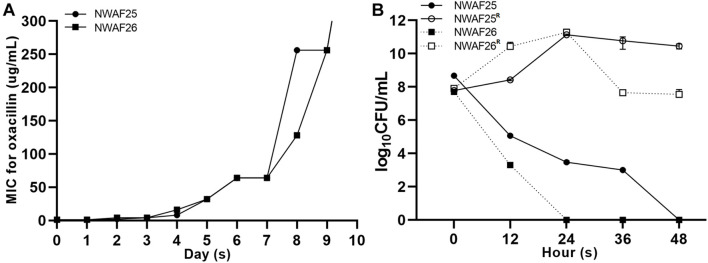
Oxacillin susceptibility assay of *Staphylococcus sciuri.* **(A)** Minimal inhibitory concentration (MIC) of oxacillin in *S. sciuri* NWAF25, NWAF26, and their oxacillin-induced derivations, starting from the 0 generation to the 10th generation. **(B)** Bactericidal time with a survival rate of 1% (MDK99) in *S. sciuri* and their derivatives. The survival rate of isolates grown in tryptic soy broth (TSB) medium at 37°C with 20 mg/L oxacillin was calculated at five time points (0, 12, 24, 36, and 48 h). Solid circle and solid square represent wild-type isolates NWAF25 and NWAF26, respectively; while hollow circle and hollow square represent the corresponding derivatives of NWAF25 and NWAF26 at 10th generation, respectively. Data are represented as means ± S.D. (error bars) from three independent experiments.

Since *mecA1* in *S. sciuri* is considered a precursor of *mecA* in MRSA (Rolo et al., 2017), the *S. sciuri* isolates were tested for β-lactam resistance. First, all five *S. sciuri* isolates were tested by PCR for the presence of *mecA* and *mecA1*. Our results showed that *mecA1* is present in three isolates (NWAF05, NWAF25, and NWAF26), while *mecA* is absent in all isolates. Next, the three *mecA1*-positive *S. sciuri* were tested for resistance against 11 common antibiotics. It was found that all three strains were resistant mainly to thiamphenicol (MIC = 16 μg/ml) and florfenicol (MIC > 256 μg/ml) (Table 3). Interestingly, although *mecA1* is present in these isolates, they were all sensitive to oxacillin (MIC = 1 μg/ml). Considering that β-lactam resistance can be induced by oxacillin in OS-MRSA strains, we treated two *S. sciuri* strains (NWAF25 and NWAF26) with oxacillin for 10 consecutive days (Pilong et al., 2016). It was found that, like *S. aureus*, oxacillin resistance can also be induced in *S. sciuri* (Figure 1A). On day 10 of induction, the MICs of oxacillin in NWAF25 and NWAF26 were observed to exceed 256 μg/ml; therefore, the induced derivatives on day 10 were named as NWAF25^R^ and NWAF26^R^, respectively (Figure 1A and Table 3). At the same time, our results showed that the MICs of β-lactam antibiotics (ampicillin and cefalotin) in the induced derivatives were increased dramatically by 32 times (Table 3). The resistance of the induced strains against vancomycin, streptomycin, and trimethoprim was observed to be slightly increased (Table 3). Heterogeneous resistance was excluded as the cause because no colonies were found from the overnight cultures of NWAF25 and NWAF26 after 48-h incubation on TSA plates supplemented with 1 μg/ml of oxacillin or higher. Next, we added 20-fold increased MIC of oxacillin to the liquid medium and measured the MDK99 value of oxacillin against the wild-type and induced derivatives of *S. sciuri.* As shown in Figure 1B, while oxacillin completely killed the wild type *S. sciuri* within 48 h, it had no inhibitory effect on the induced oxacillin-resistant isolates. We then performed 10 rounds (10 days) of reverse induction on NWAF26^R^ using oxacillin-free liquid medium. After the day 3 and day 10 of reverse induction, the strains (NWAF26^R^-R3 and NWAF26^R^-R10) still showed high resistance against three β-lactam antibiotics (oxacillin, ampicillin, and cefoxitin), indicating that β-lactam resistance remained stable within a certain period of time (Table 3).

**TABLE 3:** Minimal inhibitory concentration (MIC) of antibiotics to *Staphylococcus sciuri.*

	OXA	AMP	CEF	VAN	TMP	STR	TET	AZI	CLI	GEN	CIP	THI	FLO
NWAF05	1	2	0.5	0.5	4	2	0.5	2	2	0.25	0.25	16	>256
NWAF25	1	2	0.5	0.5	4	4	0.5	2	2	0.25	0.25	16	>256
NWAF26	1	2	0.5	0.5	4	4	0.5	2	2	0.25	0.25	16	>256
NWAF26^R^	>256	64	16	1	16	8	0.25	1	1	0.25	0.25	16	>256
NWAF26^R^-R3	256	32	16	1	8	8	0.25	1	1	0.25	0.25	16	>256
NWAF26^R^-R10	256	32	16	1	8	8	0.25	1	1	0.25	0.25	16	>256

*MIC, minimum inhibitory concentration, μg/ml; OXA, oxacillin; AMP, ampicillin; CEF, cefazolin; VAN, vancomycin; TMP, trimethoprim; STR, streptomycin; TET, tetracycline; AZI, azithromycin; CLI, clindamycin; GEN, gentamicin; CIP, ciprofloxacin; THI, thiamphenicol; FLO, florfenicol. All MIC determinations were performed by agar dilution assay.*

### The Induced β-Lactam Resistance of *Staphylococcus sciuri* Is Not Caused by *mecA1* Expression

**FIGURE 2:**
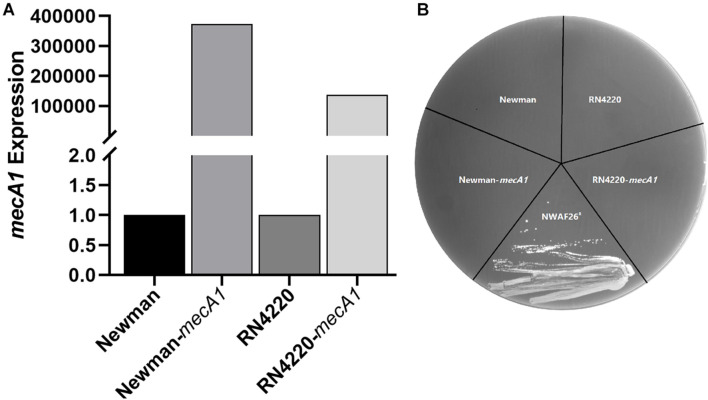
Construction and oxacillin susceptibilities of *mecA1* overexpression strains. The *mecA1* gene from *S. sciuri* NWAF26 was introduced into the methicillin sensitive *S. aureus* strains RN4220 and Newman by shuttle vector pSE1, respectively. **(A)** The expression level of *mecA1* were measured by qRT-PCR. **(B)** Oxacillin susceptibilities were determined using MH agar with 2% NaCl containing 1 mg/L oxacillin. NWAF26^R^ was taken as a positive control.

To investigate the acquisition mechanism β-lactam resistance in *S. sciuri*, we first evaluated the expression level of *mecA1* by qRT-PCR. It was found that *mecA1* expression in the resistant isolate NWAF26^R^ was slightly upregulated by 1.06 times compared with that of the wild-type NWAF26. The gene copy number of *mecA1* was found to be identical in both NWAF26^R^ and NWAF26 based on a qPCR result. Since MecR1/MecI and BlaR1/BlaI are mainly responsible for the upregulation of *mecA* expression in *S. aureus* (Pilong et al., 2016), the presence of these four regulatory genes in the three *S. sciuri* isolates (NWAF05, NWAF25, and NWAF26) was evaluated. Our results showed that *mecA* regulators were absent in all three isolates. Next, we transformed *mecA1* into two methicillin-susceptible *S. aureus* strains (Newman and RN4220), respectively, in order to construct new strains (Newman-*mecA*1 and RN4220-*mecA1*) with high *mecA1* expression levels (Figure 2A). However, both the Newman-*mecA1* and RN4220-*mecA1* strains showed sensitivity to oxacillin (MIC < 0.5 μg/ml) (Figure 2B). When the Newman-*mecA1* and RN4220-*mecA1* were subjected to antibiotic-induced passages with oxacillin, the MIC of oxacillin was observed to be unchanged. This indicated that oxacillin resistance in staphylococci is not conferred by high *mecA1* expression. So, the β-lactam resistance in *S. sciuri* may not be caused by *mecA1*.

### The Induced β-Lactam Resistance of *Staphylococcus sciuri* Is Not Caused by a Gene Mutation

**FIGURE 3:**
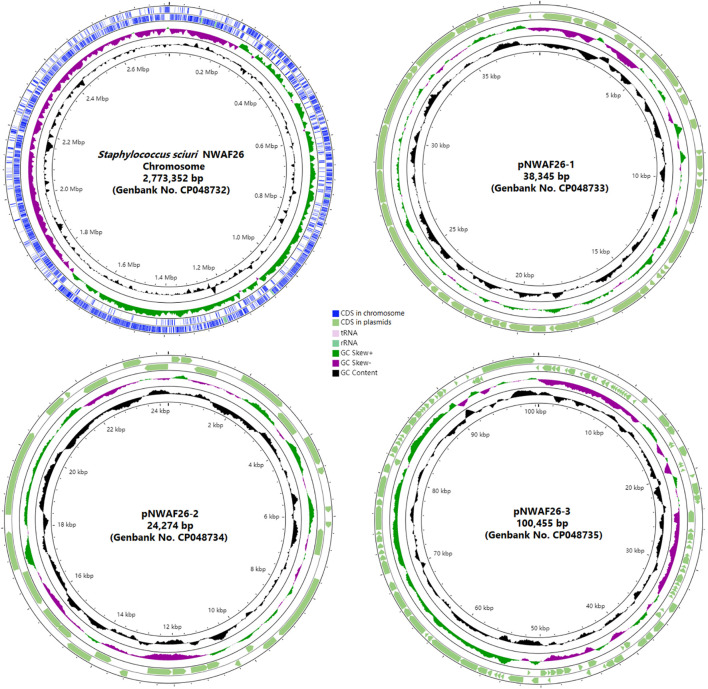
Physical map of the *S. sciuri* NWAF26 chromosome and three plasmids.

In order to further explain the mechanism of the induced β-lactam resistance in *S. sciuri*, we performed WGS and analyzed the sequences. A total of 23 contigs of wild-type NWAF26 were first obtained. Then, the gaps were closed by PCR. NWAF26 contains one chromosome (Genbank no. CP048732) with a size of 2,773 kb and three plasmids (Genbank no. CP048733, CP048734, and CP048735) with sizes of 38, 24, and 100 kb, respectively (Figure 3), that encode a total of 2,887 genes. Among the 62 published genome sequences of *S. sciuri* strains, NWAF26 has the highest chromosome similarity with *S. sciuri* B9-58B (Genbank no. CP041879, also from retail pork). CARD database prediction suggested that there are 35 antibiotic-resistant genes in NWAF26, and many are associated with efflux pump (13 genes), as well as with the resistant determinants against aminoglycoside (five genes), fosfomycin (three genes), and β-lactam (two genes as *mecA1* and *blaZ*) (Supplementary Figure 1). Among the 13 efflux pump proteins, FexA is likely the main factor of resistance against florfenicol and thiamphenicol in NWAF26 (Kehrenberg and Schwarz, 2004). In addition to *mecA1*, *blaZ* also contributes to β-lactam resistance. However, the expression of *blaZ* in NWAF26^R^ was observed to be slightly downregulated, so *blaZ* may not be the contributing factor. Consistently, sequence analysis showed that the two-component regulatory factors *mecR1/mecI* or *blaR1/blaI* or their homologs that target *mecA* are absent in *S. sciuri*.

Next, we sequenced the genome of NWAF26^R^. Comparative genomic analysis between NWAF26^R^ and NWAF26 showed that there were only two SNP variations in NWAF26^R^. Located in the transposase IS257 and IS3, both SNP variations are unable to directly affect the antibiotic resistance phenotype. Therefore, genetic mutation is not responsible for the induction of β-lactam resistance in *S. sciuri*.

### The Induced β-Lactam Resistance of *Staphylococcus sciuri* Is Caused by Thickened Cell Wall

**FIGURE 4:**
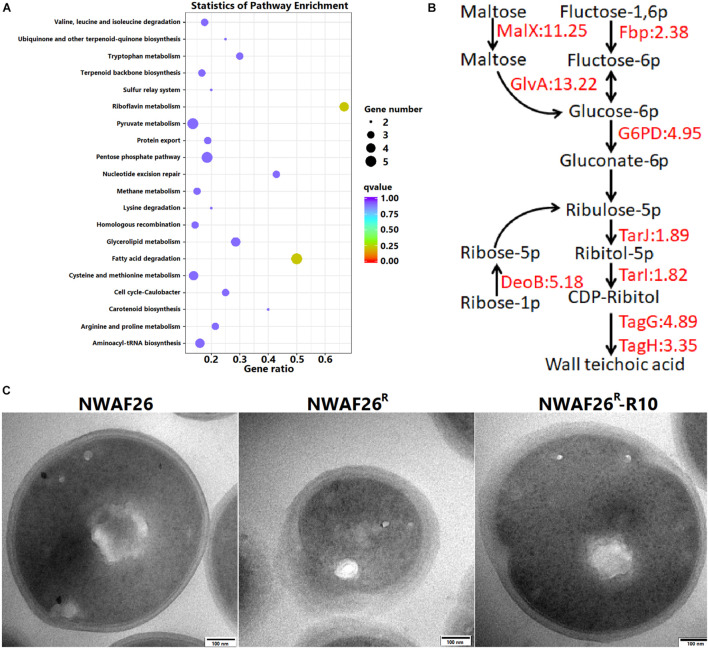
The induced β-lactam resistance of *S. sciuri* caused by the thickened cell wall. **(A)** KEGG enrichment scatter map of the upregulated differentially expressed gene in NWAF26^R^ compared with NWAF26. **(B)** The expression of genes related to wall teichoic acid (WTA) synthesis pathway was upregulated in NWAF26^R^ and highlighted with red color, the number represented the fold changes of upregulation. **(C)** Transmission electron microscopy (TEM) analysis of NWAF26 and its derivations at the stationary phase. NWAF26^ R^-R10 represents the 10th generation isolate induced from NWAF26^ R^ without oxacillin. The standard ruler is 100 nm.

We carried out a transcriptome analysis between NWAF26 and NWAF26^R^. A total of 25 DEGs were selected to validate our RNA-seq data by qRT-PCR, and the results showed a correlation index of 0.94 (Supplementary Figure 2), suggesting that our RNA-seq data were reliable. In agreement with our qRT-PCR results, transcriptome analysis showed that the expression level of *mecA1* in NWAF26^R^ was consistent to that in NWAF26 [log2 (fc) = 0.01]. We found that the expression of four genes involved in the pentose phosphate pathway in NWAF26^R^ were upregulated by 4.5-fold [log2 (fc) = 1.25∼2.60] on average (Figure 4A). In addition, the expression of MalX (EIIC), a member of phosphotransferase system that transports maltose, and GlvA, a key enzyme that metabolizes maltose-6p into glucose-6p, were upregulated by 11.2- and 13.3-fold, respectively, hence, increasing the carbon source supply for the pentose phosphate pathway (Figure 4B). The upregulation of these metabolic pathways can effectively increase the production of ribulose-5p, which can be used to synthesize ribitol-5p by alcohol dehydrogenase TarJ and then convert to CDP-ribitol by cytidylyl transferase TarI (Baur et al., 2009). Under the action of TarJ (upregulated by 1.9-fold) and TarI (upregulated by 1.8-fold), ribulose-5p is transformed into CDP-ribitol, which is used to synthesize wall teichoic acid (WTA), a component of the cell wall by TarL. Nascent WTA is translocated to the outer surface of the cell membrane by TagGH (upregulated by 4.9- and 3.4-fold, respectively), which finally leads to an increase level of WTA in the cell wall (Schirner et al., 2011; Chen et al., 2020). The expression of the two-component system (TCS) GraRS, which promotes WTA biosynthesis (Keinhrster et al., 2019), upregulated by 1.3-fold. Another analysis has shown that ltaS (Gründling and Schneewind, 2007), a key factor mediating lipoteichoic acid synthesis, is also upregulated by 2.6-fold. Meanwhile, the expression of peptidoglycan hydrolase LrgA is downregulated by 101.4-fold. Changes in the above pathways play a role in promoting the increased level of extracellular teichoic acid, which in turn increases the thickness of bacterial cell wall.

Based on the above analyses, the CWT of *S. sciuri* was evaluated by TEM. It was found that the CWT of resistant *S. sciuri* was increased greatly. The average CWT was 59.1 nm for NWAF26^R^ and 44.7 nm for NWAF26^R^-R10, which were 2.1-fold (*p* < 0.05) and 1.6-fold (*p* < 0.05) thicker than that of the wild-type NWAF26 (28.4 nm) (Figure 4C). To further confirm this finding, the cell wall mass and WTA amount were determined. The cell wall dry weight per gram of cell wall wet weight in strain NWAF26 was 10.25 ± 3.66 mg/g vs. those in strain NWAF26^R^ at 17.91 ± 4.09 mg/g (Table 4). Importantly, there were significant difference in the amount of WTA found in the cell wall of the strains, with strain NWAF26^R^ (138.31 ± 4.15) producing more WTA than strain NWAF26 (52.26 ± 3.87) (Table 4). Hence, the thickened cell wall is likely one of the contributing factors of β-lactam resistance in *S. sciuri*.

**TABLE 4:** Wall teichoic acid (WTA) in the NWAF26 and NWAF26^R^ strains.

	Cell wall dry weight (mg/g)	Amount of WTA (nmol/mg)
NWAF26	10.25 ± 3.66	52.26 ± 3.87
NWAF26^R^	17.91 ± 4.09*	138.31 ± 4.15*

Dry weight of cell wall was quantified as mg/g (mg cell wall dry weight/g cell wall wet weight); amount of WTA was expressed as nmol/mg (nmol phosphorus/mg cell wall dry weight). * represents the p-value < 0.01 based on comparisons with corresponding results for strain NWAF26 and NWAF26^R^.

### *Staphylococcus sciuri* With Increased β-Lactam Resistance Has a Slower Growth Rate

**FIGURE 5:**
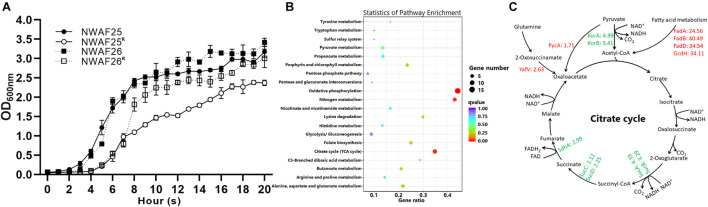
The growth rate of *S. sciuri* and their oxacillin-resistant derivations. **(A)** Growth curves of NWAF25, NWAF26, NWAF25^R^, and NWAF26^R^ isolates. Bacteria were grown at 37°C in TSB and OD_600_ values were measured at 1-h interval for 20 h. **(B)** KEGG enrichment scatter map of downregulated differentially expressed gene of NWAF26^R^ compared with NWAF26. **(C)** The expression of genes related to energy production and bacterial growth was partially downregulated in NWAF26^R^ and highlighted with green color, the number represented the fold changes of regulation.

When the cell concentration of NWAF25^R^ and NWAF26^R^ approached OD600 = 1.0, the time nodes were 2.9 h and 2.3 h later than that of their wild type, respectively, indicating that the acquisition of β-lactam resistance in *S. sciuri* results in their slower growth rate (Figure 5A). This observation can be further explained by our results from transcriptome analysis, which indicated that the expression of genes related to growth and division were downregulated in *S. sciuri* with increased β-lactam resistance. Compared with NWAF26, downregulated genes in NWAF26^R^ were found to be mainly associated with oxidative phosphorylation, citrate cycle, and nitrogen metabolism pathways (Figure 5B). A total of 15 genes of the oxidative phosphorylation pathway located in the electron transport chain complexes of I (1 gene), II (3 genes), and IV (11 genes), were downregulated by an average of 4-fold [log2(fc) = −1.27 to −2.97] (Supplementary Table 2). Meanwhile, seven genes in the citrate cycle located in the pathway from α-ketoglutarate (2-oxoglutarate) to -fumarate were downregulated by an average of 3.3-fold [log2(fc) = −1.08 to −2.32] (Figure 5C and Supplementary Table 2). Nine genes involved in the process of dissimilatory nitrate reduction to ammonium (DNRA) were downregulated by an average of 6.1-fold [log2 (fc) = −1.48 to −3.54). Downregulation of these genes can impair the electron transfer pathway by DNRA, as well as the cell ability to recycle succinate to citrate cycle through nitrate metabolism (Supplementary Table 2). Oxidative phosphorylation, citrate cycle, and nitrate metabolism are important pathways that supply energy in bacteria cells. Hence, downregulation of these pathways can inevitably decrease the energy source supply of β-lactam resistant *S. sciuri*. At the same time, the expression of *murG*, which promotes the formation of bacterial Z-ring, was downregulated by 3.3-fold, while *clpp*, an inhibitor of *ctrA* was upregulated by 2.1-fold in NWAF26^R^. These changes in *murG* and *ctrA* may result in delayed cell division in drug-resistant NWAF26^R^ (Supplementary Table 2).

On the other hand, four genes involved in the fatty acid β-oxidation pathway in NWAF26^R^ were upregulated by an average of 33.4-fold [log2(fc) = 4.62–5.34], and this can increase the yield of acetyl-Coa (Figure 5C). Furthermore, pyruvate carboxylase (PycA), which metabolizes pyruvate to oxaloacetic acid (oxaloacetate), and 2-oxoglutaramateamidase (YafV), which metabolizes asparagine to oxaloacetic acid in NWAF26^R^, were upregulated by 1.7- and 2.6-fold, respectively (Figure 5C). Additional supply of acetyl-CoA and oxaloacetic acid may supplement the deficiency of oxaloacetic acid and acetyl-CoA in the citrate cycle pathway in order to produce sufficient amount of ATP for the survival of β-lactam resistant *S. sciuri*.

### Decreased Adhesion Ability and Biofilm Thickness in Induced β-Lactam Resistant *Staphylococcus sciuri*

**FIGURE 6:**
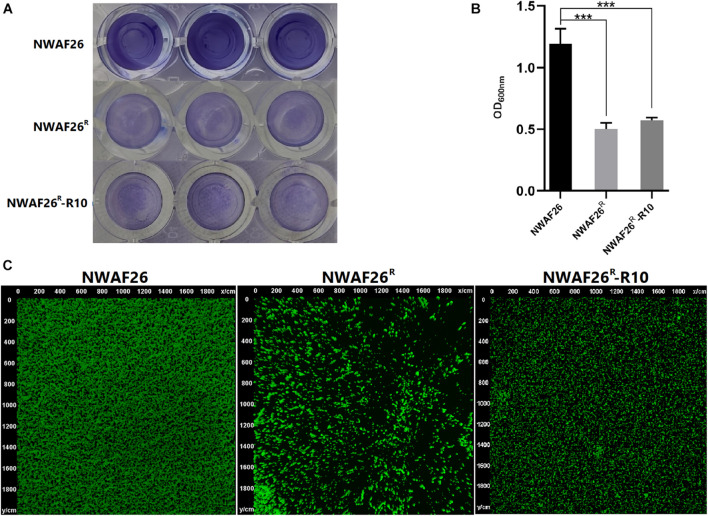
The biofilm thicknesses of NWAF26 and its derivations were determined by crystal violet staining **(A,B)** and CLSM **(C)**. The crystal violet staining was conducted by cultivating the *S. scirui* in 96-well plates at 37°C to the stationary phase, and staining with 0.5% crystal violet after being washed. The biofilm quantitation was determined by solubilizing the stained biofilms with 30% glacial acetic acid, and quantitated by measuring the OD_600_. ***p < 0.001.

Finally, we analyzed other phenotypic changes related to the survival and pathogenicity of induced β-lactam resistant *S. sciuri*. First, we evaluated the adhesion ability of *S. sciuri* to mammary gland epithelial cells MAC-T and intestinal epithelial cells Caco-2. Our results showed that, compared with that of NWAF26 (47.6% for MAC-T and 45.1% for Caco-2), the adhesion efficiency of NWAF26^R^ and NWAF26^R^-R10 to MAC-T was decreased by 3.2-fold (15.1%) and 10.4-fold (4.6%), respectively, while the adhesion efficiency of NWAF26^R^ and NWAF26^R^-R10 to Caco-2 was decreased by 3.3-fold (13.6%) and 3.1-fold (14.5%), respectively. Further transcriptome analysis showed that the expression of four adhesins in NWAF26^R^ was downregulated by an average of 4.7-fold [log2 (fc) = −1.81 to −2.67) (Supplementary Table 2). Adhesin is a key factor that promotes bacterial biofilm synthesis. Next, we measured the biofilm thickness by crystal violet staining (Figure 6A) and our results showed that, compared with that of wild type NWAF26, the biofilm thickness of NWAF26^R^ and NWAF26^R^-R10 was decreased to 42.1 and 48.1%, respectively (Figure 6B). CLSM analysis further confirmed that the biofilm thickness of β-lactam resistant *S. sciuri* was significantly thinner than that of the wild type (Figure 6C). NWAF26 forms a mature biofilm with compact structure. While the fluorescence intensity of NWAF26^R^ and NWAF26^R^-R10 is 30.2 and 41.8% of that of NWAF26, respectively. The biofilm structure of the induced derivatives is relatively loose, especially in NWAF26^R^, only a small number of bacteria adhere to the surface and no biofilm phenotype is formed.

## Discussion

In this study, three OS-MRSS isolates were isolated from 29 retail pork samples with an oxacillin MIC = 1 μg/ml, two of them were randomly selected and induced with oxacillin. On the 10th day of induction, the MICs of oxacillin to both strains exceeded 256 μg/ml. An induced derivative was selected at random and was reverse induced with an oxacillin-free liquid medium, the MIC of oxacillin to the strain slightly decreased to 256 μg/ml on day 10, indicating the β-lactam resistance can last for a long time even without the inducer oxacillin.

The primary induced β-lactam resistance mechanism in staphylococci is determined to be the increased expression of gene *mecA*, which encodes PBP2a, a penicillin-binding protein (PBP) with low affinity for β-lactam agents (Pilong et al., 2016; Chao et al., 2018). Tomasz and colleagues have transformed *S. aureus* with *mecA1*, an ortholog of *mecA*, in order to introduce β-lactam resistance (Wu et al., 2001; Antignac and Tomasz, 2009). By investigating 60 isolates of *S. sciuri* containing *mecA1* but not *mecA*, Rolo and colleagues have found that, out of six isolates that are resistant to oxacillin, five isolates have mutations in *mecA1* promoter that result in upregulated *mecA1* expression, which may contribute to β-lactam resistance in *S. sciuri* (Rolo et al., 2017). However, in this study, we observed that induced β-lactam resistance in *S. sciuri* is irrelevant to *mecA1*, because the expression level of *mecA1* in the induced resistant derivative and the wild type strain remain unchanged, and high expression of *mecA1* is unrelated to an oxacillin resistance in *S. aureus*.

There are several inexplicable questions in the previous studies related to *mecA1*. First, the expression level of *mecA1* observed is not positively correlated with the level of β-lactam resistance (Rolo et al., 2017). By detecting the *mecA1* expression level in *S. sciuri* strains K4, K5, and K7 by Western blotting, Rolo and colleagues have found that the expression of *mecA1* in K5 is significantly higher than that in K4 or K7. However, the MIC of oxacillin in K5 is only 25 μg/ml, which is much lower compared with 256 μg/ml in K4 and K7. Second, although *mecA1* is known to encode a protein with a molecular weight of 75 kDa (Genbank no. Y13052) (Duda et al., 2016), the expression product of *mecA1* has been detected with a higher molecular weight of 84 kDa by using the antibody of PBP2a (encoded by *mecA*) (Wu et al., 2001; Rolo et al., 2017; Alijani et al., 2019). This suggests that MecA (PBP2a) antibody may not be effective in detecting the expression of *mecA1*. Therefore, a different explanation of the induced resistance of *S. sciuri* in this study should be addressed except *mecA1*.

In this study, we observed that, compared with that of the wild-type, the upregulated pathways of NWAF26^R^ partly enriched in the synthesis of WTA, and that NWAF26^R^ produced more WTA than NWAF26 (138.31 vs. 52.26, Table 4). WTA constitutes up to 50% of the MRSA cell wall. We found the CWT of NWAF26^R^ was increased by 2.2-fold (Figure 4B), and that the cell wall dry mass of NWAF26^R^ was increased by 1.7-fold (Table 4). The highly hydrophilic WTA is essential to endow staphylococci with resistance against bacteriostatic molecules such as oxacillin (Brown et al., 2012; Sobhanifar et al., 2016), lysozyme (Bera et al., 2007), or fatty acids (Kohler et al., 2009). Increased cell wall WTA production is a common phenotype among MRSA isolates (Ute et al., 2013). A number of *in vitro* and *in vivo* studies have shown that WTA-deficient MRSA mutants can restore sensitivity toward β-lactams, even in the presence of PBP2A, a β-lactam resistant transpeptidase (Campbell et al., 2011; Lee et al., 2016; Bibek et al., 2019). WTA can also be used as a skeleton for peptidoglycan synthesis by helping PBP4 protein to enrich in membrane, which in turn increases the level of peptidoglycan cross-linking and CWT (Atilano et al., 2010; Brown et al., 2012). Therefore, WTA plays an important role in cell wall synthesis and β-lactam resistance of MRSA. Because the target of β-lactam agents is the cell wall, so the increased WTA synthesis observed in NWAF26^R^ is likely to be one of the main contributing factors of oxacillin resistance.

Probably due to the fact that the involved genes scattered throughout the genome, the regulation of WTA biosynthesis and modification in staphylococci is poorly understood (Keinhrster et al., 2019). A study finds that the expression of tarS is strongly upregulated by oxacillin treatment, which in turn promotes GlcNAc modification of WTA and oxacillin resistance (Brown et al., 2012). The TCS GraRS is thought to upregulate the expression of the *dltABCD* operon and increase the D-alanyl modification of WTA (Mélanie et al., 2011). In this study, the expression of GraRS was slightly increased for 1.25-fold in NWAF26^R^ compared with NWAF26. However, the role of antibiotic induction in this process is not clear. Another TCS MecR1/MecI is a good explanation for the antibiotic-induced resistance in *S. aureus* (Pilong et al., 2016). Upon β-lactam-mediated acylation, an autocatalytic cleavage occurs in the intracellular sensor domain of MecR1, which in turn leads to cleavage of MecI. This process impedes MecI binding to the promoter region and induces *mecA* transcription as well as methicillin resistance. Although *mecR1*/*mecI* is absent in the genome of the NWAF26, this mechanism could be referred to future studies.

In order to facilitate effective treatment, antibiotic sensitivity tests are often carried out to determine the antibiotic sensitivity of pathogens. The reliability of antibiotic sensitivity tests is challenged based on our results in this study, it will fail to use β-lactam antibiotics to clear OS-MRSS if the bacteria show an inducible antibiotic resistance phenotype. This phenomenon is often ignored in antibiotic sensitivity tests in clinical microbiology laboratories. Hence, further studies are required to tackle this issue.

## Data Availability Statement

The datasets presented in this study can be found in online repositories. The names of the repository/repositories and accession number(s) can be found in the article/Supplementary Material.

## Author Contributions

YC, LjZ, and HX performed the experiments, analyzed the experimental data, and wrote the manuscript. YL, XuZ, YS, XT, JX, CW, CT, LlZ, and YX performed the experiments. HX and XiZ designed the experiments, analyzed the result, and reviewed the manuscript. All authors contributed to the article and approved the submitted version.

## Conflict of Interest

The authors declare that the research was conducted in the absence of any commercial or financial relationships that could be construed as a potential conflict of interest.

## Publisher’s Note

All claims expressed in this article are solely those of the authors and do not necessarily represent those of their affiliated organizations, or those of the publisher, the editors and the reviewers. Any product that may be evaluated in this article, or claim that may be made by its manufacturer, is not guaranteed or endorsed by the publisher.

## Footnotes

1
https://card.mcmaster.ca/analyze

2
https://rast.nmpdr.org/

## Supplementary Material

Supplementary Figure 1 A survey of antibiotic-resistance genes of Nwaf26. A total of 35 genes in Nwaf26 were highly similar (more than 40%) to the reference gene sequence of the Card Database.

Supplementary Figure 2 Correlation analysis of Rna-seq data and qRt-Pcr results. The correlation index was 0.94, indicating that transcriptome data were available.
