# Supplementary figures and images for: Inducible Resistance to β-Lactams in Oxacillin-Susceptible mecA1-Positive Staphylococcus sciuri Isolated From Retail Pork
Source: Front Microbiol. 2021 Oct 20;12:721426. doi: 10.3389/fmicb.2021.721426 (PMC8564388; doi:10.3389/fmicb.2021.721426)

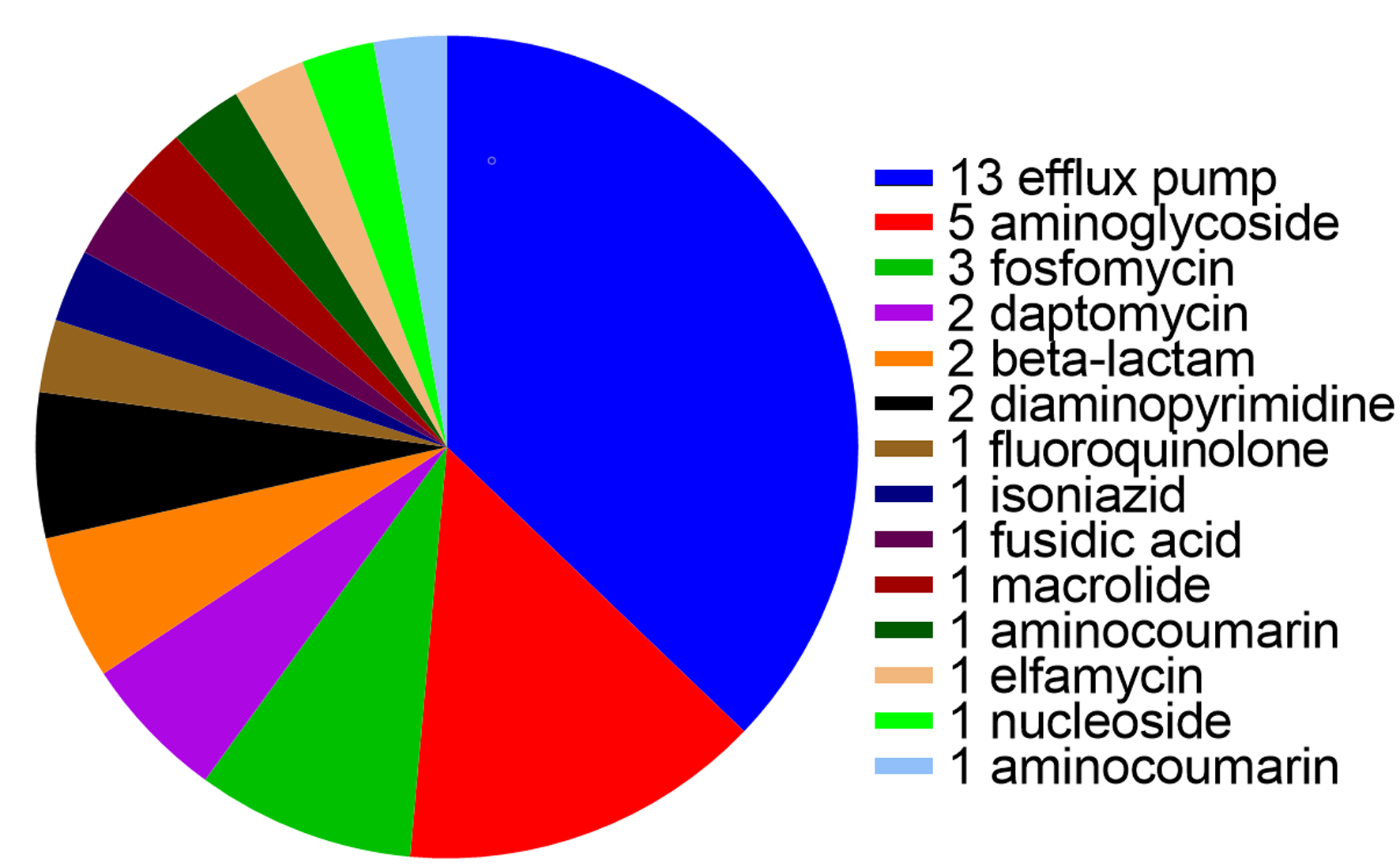

Supplement: Supplementary Figure 1 — A survey of antibiotic-resistance genes of Nwaf26. A total of 35 genes in Nwaf26 were highly similar (more than 40%) to the reference gene sequence of the Card Database. [file Image_1.TIF]

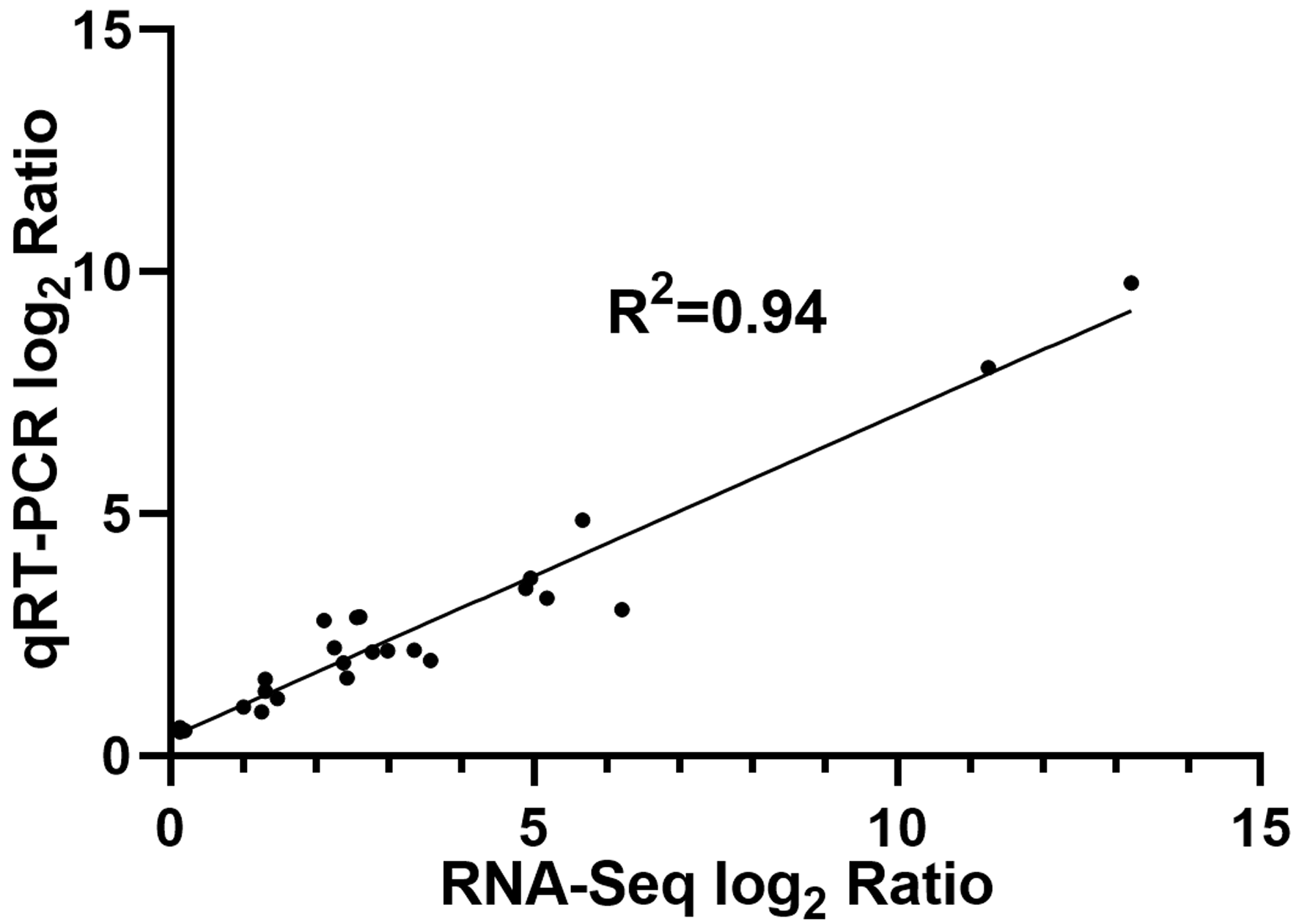

Supplement: Supplementary Figure 2 — Correlation analysis of Rna-seq data and qRt-Pcr results. The correlation index was 0.94, indicating that transcriptome data were available. [file Image_2.TIF]
