# Supplementary material for: Inducible Resistance to β-Lactams in Oxacillin-Susceptible mecA1-Positive Staphylococcus sciuri Isolated From Retail Pork
Source: Front Microbiol. 2021 Oct 20;12:721426. doi: 10.3389/fmicb.2021.721426 (PMC8564388; doi:10.3389/fmicb.2021.721426)
Supplement: Supplementary file 3 [file Table_1.DOCX]

Table S1. Sequences of primers used in this study

| Primer | Sequence (5’ to 3’) | Function |
| --- | --- | --- |
| *blaI-F* | TGAAATATCTATGGCTGAATGGGATG | Targets *blaI* |
| *blaI-R* | CAGCACTAAACTTTTCATGTCCC | Targets *blaI* |
| *blaRI-F* | AGCACTGTTTGGACTTGACCG | Targets *blaRI* |
| *blaRI-R* | CCCGTTTACTATACCTGTACCTGTT | Targets *blaRI* |
| *mecI-F* | GAAGAAATACAAATGCAAAAGGACTG | Targets *mecI* |
| *mecI-R* | CAAGTGAATTGAAACCGCCTTTG | Targets *mecI* |
| *mecRI-F* | ATCTGACACGACTTCTTCGGTTA | Targets *mecRI* |
| *mecRI-R* | GCACAAAACTTCCATCAAATCCT | Targets *mecRI* |
| *mecAI-F* | TAGTCTACAAGTAACGACCCACC | Targets *mecAI* |
| *mecAI-R* | AGGGCGGTCTGTCATTTCTACTT | Targets *mecAI* |
| *mecA-F* | ATGAAAAAGATAAAAATTGTTCCAC | Targets *mecA* |
| *mecA-R* | ATTTCTTACTGCCTAATTCGAG | Targets *mecA* |
| 27-F | AGAGTTTGATCCTGGCTCAG | Targets 16S |
| 1451-R | AAGGAGGTGATCCAGCCGCA | Targets 16S |
| qAgrD-F | CTGGTACTTCTGTTTCATCTAAA | qRT-PCR |
| qAgrD-R | GAAAGGATATTTGATACTGATTTTGA | qRT-PCR |
| qFliK-F | ATGCGAATGATAATAGTGGTGA | qRT-PCR |
| qFliK-R | TGTTCCTGCTTTTGTAGGTGTA | qRT-PCR |
| q1170-F | TACACGGCCCTAACGGAGA | qRT-PCR |
| q1170-R | AGGTAATCCTCTGTGAGCGA | qRT-PCR |
| q4345-F | GAAAAGTTATGGCTAAAAGTCGGCA | qRT-PCR |
| q4345-R | ATTGACATAGTCTCTACCAGAAGCG | qRT-PCR |
| q4240-F | TCGCAACAAAAGTAGGTTTACAAAT | qRT-PCR |
| q4240-R | TGTATCGCAAAGAAACGAGTTAGAG | qRT-PCR |
| qGraR-F | TGAGTATGGAACTGGGTGCTG | qRT-PCR |
| qGraR-R  qGraS-F  qGraS-R  qG6PD-F  qG6PD-R  qfbp-F  qfbp-R | CCGTAATCCACTCGCGCTT  TGAGTGGAGCAGAATCGACTAC  CCCTATGCCTTTTGCCATGC  AAGGGTCACTAACATCGTGCG  AATCGGTGTTGGTCGAAGGG  GAAATGCAGTATTGTCGCCG  GATTGGCGCTTATGCTGGTT | qRT-PCR  qRT-PCR  qRT-PCR  qRT-PCR  qRT-PCR  qRT-PCR  qRT-PCR |
| qdeoC-F  qdeoC-R  qdeoB-F  qdeoB-R  qglvA-F  qglvA-R  qmalX-F  qmalX-R  qtarl-F  qtarl-R  qtarJ-F  qtarJ-R  qtagG-F  qtagG-R  qtagH-F  qtagH-R  qltaS-F  qltaS-R  qsdhA-F  qsdhA-R  qcoxD-F  qcoxD-R  qqoxB-F  qqoxB-R  qsucD-F  qsucD-R  qgltB-F  qgltB-R  qclpP-F  qclpP-R  qznuB-F  qznuB-R | AGCATGTGAACTTGCTGTTGC  TGAACGGATACCACCAGAAGC  CACTTCTGCAGATCCAGTGCT  TTCGCCAACGTATGGACGTG  CACCAGGTCCACACGTTTCT  GTTACAGGCGAGTACGGACC  ACCGCTGCAGGTCCATATTC  TGGCCAACAGTACAAGCAGG  ACATCGCAAATACCTCGCTT  AGGTACGGATGATGCTTCGTT  TCTCCTAGACTTGCAGCACC  GTGCATGAATTTGATCCGACA  GCAGGTATTGCTGACAACGG  GCTCCGATAAATGGTGTACCG  TAGCTTGGATTGAAGGCGGC  ACGTGTAGCATCTAACTCGTCT  GCAACTGTTTGATCGTGTCCTT  TACCAGGTCTTAAAGGTGGCG  TTTGAGCGACAATACCGCGT  GGTGCAACAACTGGACAACA  CGAACTCTTTAGGAAGTTCAGC  TGACGCACAAGCACTTAGAGA  TATGGTCTGGGCGATCTGTG  TAACACCTGTTGGTACGGCG  GTTGCACCAGTTTCAGCGAC  CGGGTGCAACAGCACTTTTC  TTACCTGGGTTGAGTTGCCC  CTTGGCGCATTAACAGACCG  CTCCAGGTGGTAGTGTAACAGC  ACGTTTACCTTTAGCACCAGC  GGCCGCACATATTGCACTG  GCTATTGGCGCTCGTAACAG | qRT-PCR  qRT-PCR  qRT-PCR  qRT-PCR  qRT-PCR  qRT-PCR  qRT-PCR  qRT-PCR  qRT-PCR  qRT-PCR  qRT-PCR  qRT-PCR  qRT-PCR  qRT-PCR  qRT-PCR  qRT-PCR  qRT-PCR  qRT-PCR  qRT-PCR  qRT-PCR  qRT-PCR  qRT-PCR  qRT-PCR  qRT-PCR  qRT-PCR  qRT-PCR  qRT-PCR  qRT-PCR  qRT-PCR  qRT-PCR  qRT-PCR  qRT-PCR |
| q16s-F | CACACTGGAACTGAGACACG | qRT-PCR |
| q16s-R | CTGCTGGCACGTAGTTAG | qRT-PCR |
| NWAF26-F1 | GCCCAGACTATATGCTAAACTTCT | gap closing |
| NWAF26-R1 | GGCATAAATGAGATTTGCTGGAGA | gap closing |
| NWAF26-F2 | AGACGCAAATTAGATGAACATTGAA | gap closing |
| NWAF26-R2 | CATCCATTGTGATTCGTTGTCTAT | gap closing |
| NWAF26-F3 | GAGTACTGAAAGTACTTCAGAGTG | gap closing |
| NWAF26-R3 | GTACAGTATATGACCTTTATCTTCTA | gap closing |
| NWAF26-F4 | CGAGTATAATTGTTTCTTGTAGCG | gap closing |
| NWAF26-R4 | TCCCAGCTGAGCTAATTCTCC | gap closing |
| NWAF26-F5 | GTAATCTCTTGGCTTTATTCAGGA | gap closing |
| NWAF26-R5 | GATAGCTGAAGTCATGGCTTAAGT | gap closing |
| NWAF26-F6 | CTCCTCTCACAATTTTCAAGCAAA | gap closing |
| NWAF26-R6 | CGCTAGTCTCCACCATTTAAAAAT | gap closing |
| NWAF26-F7 | GGAACTGGTTATAGACTACCTTAA | gap closing |
| NWAF26-R7 | GAGTACTAAAAGTACTTCAGAGTG | gap closing |
| NWAF26-F8 | GCGTCTTACTTCTCCATTTCGTTT | gap closing |
| NWAF26-R8 | GGTGACAATGAGAAAGTGTAAATATA | gap closing |
| NWAF26-F9 | GCAGTTGTTCCAGATAGTGATAAT | gap closing |
| NWAF26-R9 | CAATTCAACTGAATTAAACATAAATTCAT | gap closing |
| NWAF26-F10 | CAATCTCAGGCACACCAACAAAAG | gap closing |
| NWAF26-R10 | CTAAAAATATCCGAAGGATATGATG | gap closing |
| NWAF26-F11 | CAAACTATTTTAGGCTACTACTAC | gap closing |
| NWAF26-R11 | CAGTTAATAACATTACAGCTGTTAAA | gap closing |
| NWAF26-F12 | CATGTCAGTGTTCGCTTAACTTG | gap closing |
| NWAF26-R12 | GTGAAACCGTGAGTTATTACATAT | gap closing |
| NWAF26-F13 | GAACTTGAACTTGTGTGATTTCTG | gap closing |
| NWAF26-R13 | CGAGATTAAACGCCTTGTACTCAA | gap closing |
| NWAF26-F14 | CTGGTTCTGTTGCAAAGTAAAAAA | gap closing |
| NWAF26-R14 | GAACTCTAAGACGTTACCACATT | gap closing |
| NWAF26-F15 | CGAAACACTTAAAGATATGACGAA | gap closing |
| NWAF26-R15 | GAGCTTACTCTAATCAGTCACTAT | gap closing |
| NWAF26-F16 | CTTGGGTGATCTCTTGGCTTTATT | gap closing |
| NWAF26-R16 | CTAAATAAAGCCAAGAGATCGTCC | gap closing |
| NWAF26-F17 | GGGAAGGATTTAATCATGAGTAAA | gap closing |
| NWAF26-R17 | GTATTCCAACCTCATCAAGTCAAG | gap closing |
| NWAF26-F18 | CAGGAATATGTCCATATAATTGTG | gap closing |
| NWAF26-R18 | TACACCCTGATTTTAGTCAAGTCC | gap closing |
| NWAF26-F19 | GTCAAGTCCATATGTTTGTGTAAT | gap closing |
| NWAF26-R19 | ATATCTTTCAGTCTTCGTGCGATA | gap closing |
| NWAF26-F20 | AGCAGTCAAGTCCATATGTTTGTG | gap closing |
| NWAF26-R20 | CTCTTGGCTTTACTGAATATGTCC | gap closing |
| NWAF26-F21 | GTCAAGTCCATATGTTTGTGTAA | gap closing |
| NWAF26-R21 | CAGTCTTCGTGCGATACACGAAT | gap closing |
| NWAF26-F22 | GGGGAATATGTCCATATAATTGTG | gap closing |
| NWAF26-R22 | GTATCGAGTCAAGTCCATATGTTT | gap closing |
| NWAF26-F23 | GCTCAATTGGCTTTATATCTTCAA | gap closing |
| NWAF26-R23 | GCTTTGGATCATTTTGCGATAACG | gap closing |

Table S2. The main differentially expressed genes between NWAF26 and NWAF26^R^

| Pathway term | Gene | Description | Protein ID | diffexp* (log2fc) | diffexp** (fold) |
| --- | --- | --- | --- | --- | --- |
| [Pentose phosphate pathway](file:///G:\Desktop\NWAF26\Manuscript%20NWAF26\%25E6%2596%25B0%25E5%25BB%25BA%25E6%2596%2587%25E4%25BB%25B6%25E5%25A4%25B9%20(2)\KEGG%25E5%2588%2586%25E6%259E%2590\KEGG\diff_1\UP\src\ko00030.html) | G6PD | glucose-6-phosphate dehydrogenase | QDR64060.1 | 2.31 | 4.95 |
|  | fbp | fructose-1,6-bisphosphatase | QDR65707.1 | 1.25 | 2.38 |
|  | deoC | deoxyribose-phosphate aldolase | QDR63482.1 | 2.50 | 5.67 |
|  | deoB | phosphopentomutase | QDR63484.1 | 2.37 | 5.18 |
|  | glvA | 6-phospho-alpha-glucosidase | QDR65083.1 | 3.73 | 13.22 |
|  | malX | PTS alpha-glucoside transporter subunit IIBC | QDR65084.1 | 3.49 | 11.25 |
|  | tarI | cytidylyl transferase | QDR65239.1 | 0.32 | 1.25 |
|  | tarJ | NADPH-dependent alcohol dehydrogenase | QDR65238.1 | 0.56 | 1.47 |
|  | tagG | teichoic acids export ABC transporter permease | QDR64563.1 | 2.29 | 4.89 |
|  | tagH | teichoic acids export ABC transporter ATP-binding subunit | QDR64564.1 | 1.74 | 3.35 |
|  | ltaS | lipoteichoic acid synthase | QDR64864.1 | 1.38 | 2.60 |
|  | lrgA | antiholin-like murein hydrolase modulator | [QDR65959.1](https://www.ncbi.nlm.nih.gov/protein/1710313348) | -6.66 | -101.39 |
| [Oxidative phosphorylation](file:///G:\Desktop\NWAF26\Manuscript%20NWAF26\新建文件夹%20(2)\KEGG分析\KEGG\diff_4\DOWN\src\ko00190.html) | sdhA | succinate dehydrogenase flavoprotein subunit | QDR64442.1 | -1.58 | -2.99 |
|  | petA | ubiquinol-cytochrome c reductase iron-sulfur subunit | QDR64100.1 | -2.42 | -5.34 |
|  | petB | cytochrome b6 | QDR64101.1 | -2.96 | -7.80 |
|  | petC | cytochrome C oxidase Cbb3 | QDR64102.1 | -1.81 | -3.51 |
|  | coxD | cytochrome B6 | QDR64469.1 | -1.28 | -2.43 |
|  | coxC | cytochrome (ubi)quinol oxidase subunit III | QDR64470.1 | -1.84 | -3.57 |
|  | coxA | cytochrome c oxidase subunit I | QDR64471.1 | -2.05 | -4.14 |
|  | coxB | cytochrome c oxidase subunit II | QDR64472.1 | -2.31 | -4.97 |
|  | ctaD | b(o/a)3-type cytochrome-c oxidase subunit 1 | QDR64119.1 | -2.15 | -4.45 |
|  | ctaC | RS06530cytochrome B5 | QDR64120.1 | -2.97 | -7.85 |
|  | qoxA | cytochrome aa3 quinol oxidase subunit II | QDR64538.1 | -1.67 | -3.18 |
|  | qoxB | cytochrome aa3 quinol oxidase subunit I | QDR64539.1 | -1.36 | -2.56 |
|  | qoxC | cytochrome aa3 quinol oxidase subunit III | QDR64540.1 | -1.27 | -2.41 |
|  | ctaB | protoheme IX farnesyltransferase | QDR64473.1 | -2.47 | -5.54 |
|  | ctaA | heme A synthase | QDR64474.1 | -1.56 | -2.95 |
| [Citrate cycle (TCA cycle)](file:///G:\Desktop\NWAF26\Manuscript%20NWAF26\新建文件夹%20(2)\KEGG分析\KEGG\diff_4\DOWN\src\ko00020.html) | sdhA | succinate dehydrogenase flavoprotein subunit | QDR64442.1 | -1.58 | -2.99 |
|  | korB | 2-oxoacid:acceptor oxidoreductase subunit beta | QDR64277.1 | -1.77 | -3.41 |
|  | korA | 2-oxoacid:acceptor oxidoreductase subunit alpha | QDR64278.1 | -2.32 | -4.99 |
|  | sucA | 2-oxoglutarate dehydrogenase E1 component | QDR64145.1 | -2.13 | -4.39 |
|  | sucB | 2-oxoglutarate dehydrogenase E2 component | QDR64146.1 | -1.72 | -3.29 |
|  | sucD | succinyl-CoA synthetase alpha subunit | QDR64346.1 | -1.17 | -2.25 |
|  | sucC | succinyl-CoA synthetase beta subunit | QDR64347.1 | -1.08 | -2.12 |
| [Nitrogen metabolism](file:///G:\Desktop\NWAF26\KEGG分析\KEGG\diff_4\DOWN\src\ko00910.html) | ncd2 | nitronate monooxygenase | QDR65335.1 | -2.53 | -5.77 |
|  | cobA | uroporphyrinogen-III C-methyltransferase | QDR65449.1 | -3.00 | -8.00 |
|  | nirD | nitrite reductase small subunit NirD | QDR65450.1 | -3.51 | -11.40 |
|  | nirB | NAD(P)/FAD-dependent oxidoreductase | QDR65451.1 | -3.49 | -11.26 |
|  | unknown | GNAT family N-acetyltransferase | QDR65441.1 | -3.54 | -11.65 |
|  | glnA | type I glutamate--ammonia ligase | QDR64256.1 | -2.89 | -7.39 |
|  | gltD | glutamate synthase subunit beta | QDR65300.1 | -2.19 | -4.55 |
|  | gltB | glutamate synthase large subunit | QDR65301.1 | -1.48 | -2.78 |
|  | moaE | molybdenum cofactor biosynthesis protein MoaE | QDR63389.1 | -1.80 | -3.47 |
|  | rpoD | RNA polymerase sigma factor | QDR63994.1 | -2.18 | -4.54 |
|  | thiN | thinamine pyrophosphokinase | QDR64376.1 | -1.82 | -3.54 |
|  | murG | beta-N-acetylglucosaminyltransferase | QDR64141.1 | -1.72 | -3.29 |
|  | clpP | TP-dependent Clp protease subunit | QDR64810.1 | 1.08 | 2.11 |
| [Fatty acid degradation](file:///G:\Desktop\NWAF26\Manuscript%20NWAF26\%E6%96%B0%E5%BB%BA%E6%96%87%E4%BB%B6%E5%A4%B9%20(2)\KEGG%E5%88%86%E6%9E%90\KEGG\diff_4\UP\src\ko00071.html) | ydiF | Acyl CoA:acetate/3-ketoacid CoA transferase | QDR65561.1 | 5.97 | 62.54 |
|  | fadD | acyl--CoA ligase | QDR65562.1 | 5.11 | 34.54 |
|  | gcdH | glutaryl-CoA dehydrogenase | QDR65563.1 | 5.09 | 34.11 |
|  | fadB | 3-hydroxyacyl-CoA dehydrogenase | QDR65564.1 | 5.34 | 40.49 |
|  | fadA | Acetyl-CoA acetyltransferase | QDR65565.1 | 4.62 | 24.56 |
|  | pycA | Pyruvate carboxylase | QDR64475.1 | 0.78 | 1.71 |
|  | yafV | 2-oxoglutaramate amidase | QDR63624.1 | 1.39 | 2.63 |
| adhesins | unknown | YSIRK-type signal peptide-containing protein | QDR64123.1 | -2.33 | -5.03 |
|  | unknown | LPXTG cell wall anchor domain-containing protein | QDR65947.1 | -1.94 | -3.82 |
|  | unknown | YSIRK-type signal peptide-containing protein | QDR65953.1 | -1.81 | -3.51 |
|  | unknown | YSIRK-type signal peptide-containing protein | QDR66052.1 | -2.67 | -6.36 |
| Iron binding | ftn | ferritin | QDR63671.1 | 2.55 | 5.85 |
|  | yjbI | hemoglobin | QDR64619.1 | 0.83 | 1.78 |
| Zinc transport | fur | Zn2+ uptake transcription repressor protein | QDR64003.1 | 2.85 | 7.23 |
|  | znuA | Zn2+ transport system, substrate-binding component | QDR65776.1 | -0.27 | -1.20 |
|  | znuB | Zn2+ transport system, permease component | QDR65777.1 | -1.84 | -3.58 |
|  | znuC | Zn2+ transport system, ATPase component | QDR65778.1 | -1.68 | -3.21 |
|  | czcD | Co/Zn/Cd efflux system component | QDR65829.1 | 2.25 | 4.76 |
| [Riboflavin metabolism](file:///G:\Desktop\NWAF26\Manuscript%20NWAF26\%25E6%2596%25B0%25E5%25BB%25BA%25E6%2596%2587%25E4%25BB%25B6%25E5%25A4%25B9%20(2)\KEGG%25E5%2588%2586%25E6%259E%2590\KEGG\diff_4\UP\src\ko00740.html) | ribD | Bifunctional diaminohydroxyphosphoribosylaminopyrimidine deaminase/5-amino-6-(5-phosphoribosylamino)uracil reductase | QDR63791.1 | 2.60 | 6.05 |
|  | ribE | riboflavin synthase | QDR63792.1 | 2.45 | 5.47 |
|  | ribA | Bifunctional 3,4-dihydroxy-2-butanone-4-phosphate synthase/GTP cyclohydrolase II | QDR63793.1 | 2.45 | 5.46 |
|  | ribH | 6,7-dimethyl-8-ribityllumazine synthase | QDR63794.1 | 2.00 | 4.01 |
|  | blaZ | penicillin-hydrolyzing class A beta-lactamase | QDR65424.1 | -0.34 | -1.27 |

*: The log2 Fold Change value of differentially expressed genes between NWAF26 and NWAF26^R^.

**: The Fold Change value of differentially expressed genes between NWAF26 and NWAF26^R^.
